# Viral Metagenomics on Blood-Feeding Arthropods as a Tool for Human Disease Surveillance

**DOI:** 10.3390/ijms17101743

**Published:** 2016-10-19

**Authors:** Annika Brinkmann, Andreas Nitsche, Claudia Kohl

**Affiliations:** Robert Koch Institute, Centre for Biological Threats and Special Pathogens, Seestrasse 10, Berlin 13353, Germany; NitscheA@rki.de (A.N.); KohlC@rki.de (C.K.)

**Keywords:** viral metagenomics, hematophagous arthropods, blood-feeding arthropods, vector-enabled metagenomics, xenosurveillance, emerging infectious diseases, virus surveillance, mosquitoes

## Abstract

Surveillance and monitoring of viral pathogens circulating in humans and wildlife, together with the identification of emerging infectious diseases (EIDs), are critical for the prediction of future disease outbreaks and epidemics at an early stage. It is advisable to sample a broad range of vertebrates and invertebrates at different temporospatial levels on a regular basis to detect possible candidate viruses at their natural source. However, virus surveillance systems can be expensive, costly in terms of finances and resources and inadequate for sampling sufficient numbers of different host species over space and time. Recent publications have presented the concept of a new virus surveillance system, coining the terms “flying biological syringes”, “xenosurveillance” and “vector-enabled metagenomics”. According to these novel and promising surveillance approaches, viral metagenomics on engorged mosquitoes might reflect the viral diversity of numerous mammals, birds and humans, combined in the mosquitoes’ blood meal during feeding on the host. In this review article, we summarize the literature on vector-enabled metagenomics (VEM) techniques and its application in disease surveillance in humans. Furthermore, we highlight the combination of VEM and “invertebrate-derived DNA” (iDNA) analysis to identify the host DNA within the mosquito midgut.

## 1. Surveillance of Emerging Viruses

**Figure 1:**
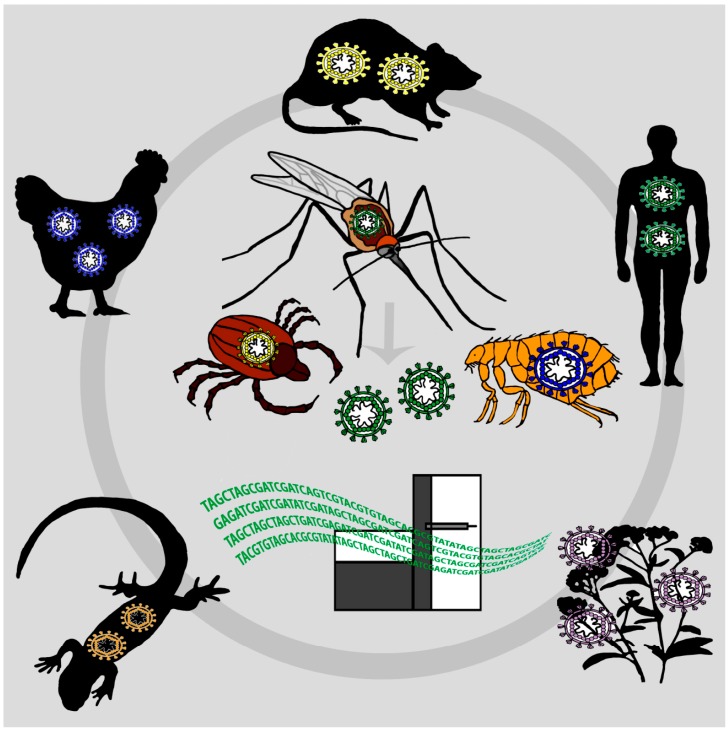
Vector-enabled metagenomics (VEM) as a tool for virus surveillance: Hematophagous arthropods feed on a wide range of hosts. The blood meals of mosquitoes, ticks, fleas, flies and midges therefore reflect the viral diversity of the host on which the arthropod has fed on, including mammals, birds, reptiles, humans and plants. Sequencing of such arthropods’ blood meals with next-generation sequencing technologies can shed light on the viral diversity of several host species within a distinct region and can be used as a tool for viral disease surveillance.

The recent epidemic of Ebola virus in Africa as well as the emergence of a hitherto unknown virus known as Middle East respiratory syndrome coronavirus (MERS-CoV), Bas-Congo virus in central Africa or of severe fever with thrombocytopenia syndrome virus (SFTSV) in China have repeatedly shown the global impact of emerging infectious diseases (EIDs) on economics and public health [1,2,3,4]. These EIDs, more than 60% of which are of zoonotic origin, are globally emerging and re-emerging with increased frequency [5]. Surveillance and monitoring of viral pathogens circulating in humans and wildlife and the identification of EIDs at an early stage is challenging. Many potential emerging viruses of concern might already be infecting humans or wildlife but await their detection by disease surveillance. In remote and underdeveloped regions of the world, often no attention is paid towards possible infectious disease cases until a threshold of serious cases and deaths appears in a cluster and certain epidemic properties are reached [3]. Some viruses might just be overlooked at population levels until they spread or re-emerge and become epidemic in another region or time. An effective strategy in virus surveillance would need to survey simultaneously a wide range of viral types in a large number of human and wildlife individuals in order to detect viruses before spreading. For example, the EcoHealth Alliance within the surveillance program PREDICT seeks to identify new EIDs before they emerge or re-emerge. Therefore, wildlife animals that are likely to carry viruses with zoonotic potential, e.g., bats, rodents, birds and primates, are sampled frequently [6,7]. However, collecting swabs or blood from sufficient numbers of wildlife individuals and the subsequent identification of viruses is challenging. The solution for overcoming this challenge might be presented by the disease vector itself. Blood feeding arthropods feed on blood from a wide range of hosts including humans, mammals and birds [8]. Therefore, they act as “syringes”, sampling numerous vertebrates and collecting the viral diversity over space, time and species. Xenosurveillance and vector-enabled metagenomics (VEM) are surveillance approaches that can exploit mosquitoes to capture the viral diversity of the animal, human or plant host the mosquito has fed on (Figure 1). Xenosurveillance, a term introduced by Brackney et al., refers to the identification of viral pathogens from total nucleic acids extracted from mosquito blood meals, either by next-generation sequencing (NGS) or conventional PCR assays [9]. Recent developments in NGS and viral metagenomics, which is the shotgun sequencing of viral nucleic acids extracted from purified virus particles, offer great opportunities for the characterization of the complete viral diversity in an organism or a population [10]. VEM, a technique used to sequence purified viral nucleic acids directly from insect vectors, has already been used to detect both animal and plant viruses circulating in vectors [11,12]. This review summarizes findings from xenosurveillance efforts as well as VEM studies using mosquitoes, since both approaches combine sampling of multiple individuals of blood-feeding arthropods with the high-throughput properties of NGS.

## 2. Mosquitoes as “Flying Syringes” for Virus Surveillance

To our knowledge, there are only few mosquito virome studies in the database as yet [11,13,14,15,16,17,18,19]. In this review, mosquito viruses are defined as follows: mosquito-infecting viruses and mosquito-borne viruses. Mosquito-infecting viruses infect mosquitoes naturally and replicate in mosquito cells in vitro, but do not replicate in vertebrate cells or infect humans or other vertebrates [20]. Novel NGS technologies have led to the discovery of numerous mosquito-infecting viruses, most of them within the families *Parvoviridae*, *Flaviviridae*, *Togaviridae*, *Rhabdoviridae*, *Bunyaviridae*, *Reoviridae*, *Mesoniviridae*, *Tymoviridae* and *Birnaviridae* [11,17]. In contrast, mosquito-borne viruses replicate within the mosquito, but can also be transmitted biologically to vertebrates and infect vertebrate cells [21]. The majority of mosquito-borne viruses belong to the families *Togaviridae*, *Flaviviridae* and *Bunyaviridae*, comprising highly pathogenic viruses such as Dengue virus, Chikungunya virus, Yellow fever virus, Japanese encephalitis virus, West Nile virus and Rift Valley fever virus [22,23].

Most studies on mosquito viruses focus on mosquito-infecting viruses or mosquito-borne viruses, neglecting the presence of vertebrate viruses originating from the blood of the mosquitoes’ host that have accumulated in the mosquitoes’ intestine during blood feeding. Shi et al. give an example of such viruses in the metagenomic survey on viral abundance in mosquitoes (*Culex tritaeniorhynchus*, *Anopheles sinensis*, *Armigeres subalbatus* and *Culex fatigans*) from Hubei province in China [13]. 3.6% of all viruses found in the metagenomics survey were neither mosquito-infecting nor mosquito-borne, but assumed to be of vertebrate origin. These viruses were closely related to torque teno sus virus 1 (family *Anelloviridae*, genus Iotatorquevirus) which is widely distributed in pigs [24]. In addition, sequences belonging to the genus Parvovirus were identified that were closely related to porcine parvovirus. Mosquitoes were collected from cowsheds and pigpens located in different areas of Hubei province. Since mosquitoes are not known to be vectors of torque teno sus virus 1 and porcine parvovirus, it is likely that the mosquito had ingested viremic blood during blood feeding on diseased pigs.

Barbazan et al. reported a first targeted approach for vectored vertebrate virus detection using mosquito blood meals. They collected blood-engorged mosquitoes near a poultry farm during an outbreak of avian influenza in Thailand and found H5N1 virus sequences in the mosquito pools tested by using RT-PCR [25].

The combination of using mosquitoes as “flying syringes” and NGS for virus surveillance was introduced by Ng and others using the term vector-enabled metagenomics (VEM) [11]. The group sequenced mosquito samples from different sites in California and found a broad range of already known and highly diverse DNA viruses, including anelloviruses, herpesviruses, poxviruses and papillomaviruses. These viruses infect a wide range of hosts including humans, mammals and birds and are not assumed to be transmissible by mosquitoes. Viruses of possible human origin were human papillomavirus 23 (HPV23), human herpesvirus 1 and human papillomavirus type 112 (HPV112). Human herpesvirus 1 is a neurotropic α-herpesvirus that causes infections of epithelia lining oral mucosa, conjunctiva, cornea and skin [26]. Papillomaviruses can infect epithelial cells of the skin or inner lining of tissues [27,28]. More than 80% of healthy human skin is asymptomatically infected with different papillomaviruses [29]. It is possible that both papillomaviruses and herpesvirus have been transferred from the human skin to the mosquito during feeding. Viruses belonging to the *Anelloviridae* can be identified in blood [30]. Anelloviruses infect humans, vertebrates and marine mammals [31,32]. It is possible that anelloviruses in the mosquito virome were ingested from the viremic hosts during blood feeding.

### 2.1. Feasibility Studies

If VEM and such approaches will be used to survey human and animal viruses circulating in a given area, it is of importance to understand how long viruses last in the mosquito’s blood meal. Following the introduction of VEM by Ng and others, several studies have shown that both DNA and RNA viruses are still detectable in the mosquito blood meal at least 24 h after ingestion. Kading et al. used *Aedes albopictus* mosquitoes as flying “biological syringes” to draw blood for virus titer determinations in small lab-housed vertebrates [33]. The lab animals (avian: chicks *Gallus gallus*, house sparrows *Passer domesticus*; mammals: hamsters *Mesocricetus auratus*) were experimentally infected with West Nile virus (*Flaviviridae*) and Highlands J virus (*Togaviridae*). Blood samples were recovered from the mosquitoes’ blood meals after feeding on the mentioned lab animals and compared to blood drawn by syringe. Virus titers derived from these two methods were not significantly different. The technique has also been successfully used on small reptiles before, but was not compared to standard methods and viremia titers were not measured [34].

In a second feasibility study to use mosquito blood meals for virus detection with a novel approach called xenosurveillance, Grubaugh and others developed a laboratory model to explore variables influencing detection of human immunodeficiency virus 1, West Nile virus, Pirital virus, Lassa virus and Chikungunya virus in blood-fed *Anopheles gambiae* mosquitoes [9]. The pathogen load and the stability of viral nucleic acids present in the mosquitoes’ blood meals were determined at different time points after blood feeding by qRT-PCR and NGS. They showed that viral nucleic acids could be detected from blood-fed *Anopheles gambiae* mosquitoes for up to 24 h post feeding. Furthermore, RNA virus recovery from mosquitoes that were allowed to feed artificially on a membrane feeding apparatus and from mosquitoes fed on hamsters with active replicating infections did not differ significantly.

To assess the feasibility of xenosurveillance for the detection of human pathogens in real settings, indoor resting, blood-fed *Anopheles gambiae* mosquitoes were collected in villages in northern Liberia. Using NGS, the research group found human Epstein-Barr virus (EBV), a highly prevalent gamma-herpesvirus that infects B-lymphocytes [35,36]. It is therefore likely that EBV was ingested from the human white blood cells in blood the mosquito fed on. In one pooled mosquito blood sample, canine distemper virus (CDV) was found, and, even more interestingly, genetic material of canine origin in the same pool.

A third feasibility study for using mosquito blood meals as a tool for human and animal virus surveillance was conducted by Yang and others [37]. The study explored the viral nucleic acid stability at different time points after mosquito ingestion. *Anopheles stephensi* mosquitoes were fed with Dengue virus-infected blood, and the efficiency of recovering viral RNA was analyzed after serial time points by qRT-PCR and NGS. Results showed that viral Dengue RNA in the blood meal is gradually degraded but still detectable 24 h after blood feeding.

### 2.2. Other Hematophagous Animals for VEM

Since various blood-feeding animals may accumulate viruses of interest, VEM surveillance efforts should not be limited to mosquitoes exclusively. Here we summarize several groups of hematophagous arthropods and leeches that might be useful for surveillance purposes.

#### 2.2.1. Ticks

Ticks (class *Arachnida*, subclass *Acari*) are a source of many highly pathogenic vector-borne diseases such as Crimean-Congo hemorrhagic fever virus (CCHFV) and tick-borne encephalitis virus (TBEV) [38,39,40]. To our knowledge, only a few studies have been published on tick metagenomes [41,42,43,44]. Blood digestion in ticks is a slow, intracellular process. Unlike mosquitoes, some tick species feed slowly for days on their host and concentrate their blood meals by excreting water and sodium ions [45]. It has been shown that host DNA is protected from degradation and can be recovered from the tick after feeding [46,47]. The metagenomic analysis of *Rhipicephalus* spp. ticks from Yunnan, China, revealed the presence of a virus belonging to the *Anelloviridae* that was closely related to torque teno canis virus, suggesting a vertebrate origin of the virus [42]. Anelloviruses, which are usually found in the blood of vertebrates, were also found in mosquito viromes [11,13,30]. For VEM, blood-engorged ticks could be sampled directly from the host body or be collected with flagging techniques [48]. However, success of VEM depends on the tick species and stage of life as some tick species feed on a single host during their whole life cycle and do not seek a new host after detachment [45].

#### 2.2.2. Midges

Biting midges (Diptera, *Ceratopogonidae*, *Culicoides* Latreille), small blood-sucking flies, have been found in almost all parts of the world in all environments [49]. Female midges feed on a variety of hosts, and therefore a diversity of plant, animal and human viruses can be identified in the virome [50]. They transmit various culicoides-borne arboviruses, e.g., Schmallenberg virus in European ruminants, Bluetongue virus, Oropouche virus and African horse sickness virus [51,52,53]. Non-vertebrate viruses, such as faustovirus-like asfarviruses, have also been found by metagenomic analyses in hematophagous biting midges and their vertebrate hosts [54].

#### 2.2.3. Carrion Flies

Wild-caught carrion flies (*Calliphora nigribarbis* and *Aldrichina grahami*) sampled near poultry farms have been used for the detection of H5N1 virus [55]. Also, Newcastle disease virus has been detected in carrion flies and correlated to a virus outbreak in a poultry farm nearby [56]. It is suspected that the viruses have been transported mechanically by the flies from chicken droppings and secretions or contaminated surfaces or have been actively ingested by the flies. Other examples of mechanically transmitted viruses by flies are the transmission of turkey coronavirus and rotavirus by domestic houseflies (*Musca domestica*) [57,58]. Furthermore, carrion flies have been used for the targeted detection of mammalian species DNA and biodiversity [59,60]. Since carrion flies feed on many vertebrate carcasses, it is feasible to use them for the detection of viruses from deceased animals.

#### 2.2.4. Bed Bugs

Common bed bug (*Cimex lectularius* and *Cimex hemipterus*, family *Cimicidae*) infestations are increasing worldwide also in industrialized countries [61]. To our knowledge, there are no virome analyses of bed bugs. Since they live in close proximity to humans and are nocturnal blood feeders, bed bugs might be easily sampled and used for VEM. Bed bugs are wingless, obligate hematophagous ectoparasites and feed on bats, birds and mammals. Bed bugs are suspected of transmitting infectious agents [62]. Hepatitis B virus (HBV) has been detected in bed bugs but no biologic replication could be observed [61,63]. Furthermore, it has been demonstrated that human immunodeficiency virus (HIV) can be detected for eight days after ingestion of concentrated virus in experimental blood meals, with no active replication in bed bugs [64].

#### 2.2.5. Fleas

Fleas (order Siphonaptera) are hematophagous insects which feed on mammals, including humans, and birds. They are known to transmit feline leukemia virus and myxoma virus [65,66]. To our knowledge, there are no virome or metagenomic analyses on fleas, and further studies are necessary to prove the concept of VEM on fleas.

#### 2.2.6. Bat Flies

Bat flies (superfamily *Hippoboscoidea*, families *Streblidae* and *Nycteribiidae*) are specialized ectoparasititic insects only associated with bats [67]. They permanently live on the bat fur and wing membranes where they feed on the bat’s blood. Bats are reservoir hosts for several emerging and re-emerging viral pathogens [68]. It is possible that also bat flies transfer such viruses among bats or bat workers, a fact that would make them excellent targets for disease monitoring. To our knowledge, there are no studies on bat fly viruses or bat fly metagenomics.

#### 2.2.7. Leeches (phylum Annelida)

Leeches belong to the phylum Annelida. They can be found in freshwater, terrestrial and marine environments [69]. They feed on various animals, among them birds and reptiles, and can therefore ingest DNA and viruses from various hosts [69,70,71]. The medicinal leech (*Hirudo medicinalis*) is able to take up to 15 mL of blood from its host [71]. Upon feeding, leeches store concentrated blood for several months. Host DNA ingested from the leech can be detected for more than four months, and RNA and DNA viruses (bovine parvovirus, feline calicivirus, equine arteritis virus and equine herpesvirus type 1) absorbed by terrestrial leeches were shown to remain infectious within the leech for up to six months [71,72,73]. It is feasible that non-infectious DNA or RNA can be detected from the leech for even longer periods. However, not all species of leeches are blood feeding and their distribution is scarce. 90% of all leeches feed solely on decomposing bodies and open wounds of amphibians, reptiles, waterfowl, fish and mammals [74]. Most species of hematophagous terrestrial leeches live in tropical rainforests of Asia, Madagascar and Australia, areas with sparse human population [75]. Therefore, sampling of leeches for human virus surveillance is challenging.

#### 2.2.8. Lice

Lice are wingless arthropods, which live as permanent ectoparasites of birds, mammals and humans. No zoonotic transmission of viruses from lice has been reported, but VEM has been shown successfully for human body lice (*Pediculus humanus corporis*) engorged in vitro with artificially infected human blood [76].

## 3. Combining VEM with iDNA

As reported by Grubaugh et al., the detection of a canine distemper virus from a mosquito blood meal by using VEM coincided with the detection of canine host DNA from the same sample [9]. This combines VEM with invertebrate-derived DNA (iDNA), a term designating the genetic material ingested by invertebrates feeding on vertebrates [77]. Several studies have shown that iDNA from a variety of vertebrate species can be identified from field-caught animals, including mosquitoes [78,79], carrion flies [59,60,80], biting midges [81], leeches [71,82] and ticks [83]. For the identification of host DNA from arthropod blood meals, PCR-based or serological techniques are traditionally used [84,85,86,87,88]. Recently, a novel high-throughput sequencing approach for identification of host DNA from blood meals was developed [89]. NGS allows the characterization and quantification of the blood meal without the use of host-specific primers. Analyses of the pre-amplified and sequenced 16S ribosomal RNA genes revealed that 83.7% of all mosquitoes in the study were feeding on one host species exclusively, with human, dog and pig as the most common hosts. Furthermore, analysis of the human hypervariable region I might determine if mosquitoes fed on more than one person. All these findings support that the principle of iDNA can be used to link viruses identified in mosquitoes and other blood-feeding arthropods with their host.

## 4. Mosquito Sampling: Practical Considerations

There are more than 3500 species of mosquitoes described on every continent except for Antarctica [90]. Mosquito sampling is easy and can be conducted passively with light- or CO_2_-baited traps [91]. Active sampling can involve collecting mosquitoes with hand nets or aspirators [92]. The traditional gold standard for sampling mosquitoes is the human landing catch (HLC) method. HLC requires a volunteer sitting with lower legs exposed to collect mosquitoes that come to feed on them [93]. Despite ethical and safety considerations, HLC is the simplest and most effective method for mosquito sampling. For surveillance of human viruses, resting mosquitoes should be sampled near or in human housing, including house walls, ceilings and bed nets. However, sampling of mosquitoes depends on the mosquitoes’ activity in different regions over the year. Seasonal abundance of most mosquito populations decreases during the dry season and peaks during the wet season [94,95]. In temperate and cold regions mosquitoes die, hibernate or enter diapause; hence sampling cannot be provided throughout the year [96,97]. For some species, mosquitoes are highly host specific, and therefore sampling can represent the virome and abundance of the preferred host [98]. Sampling strategies for VEM should focus on geographic regions where the risk of emergence or re-emergence of viral diseases is high, and include antropophilic as well as zoophilic mosquitoes. Mosquito species of the genus *Anopheles* are abundant in all geographic emerging disease “hotspots” specified by Jones et al., including the northeastern United States, central America, western Europe, tropical Africa and Southeast Asia [5,99]. Mosquito species such as *Anopheles quadrimaculatus* in the eastern part of the United States, *Anopheles atoparvus* in most parts of western Europe or *Anopheles gambiae* in Africa can be collected in or close to human habitats. For the surveillance of human viruses with VEM, *Anopheles quadrimaculatus* mosquitoes can be collected from human dwellings, where the human blood feeding rate can reach 93% [100]. Adult females of *Anopheles atoparvus* preferably feed on domestic animals but bite humans readily. They can be collected from animal shelters, households or other kinds of human-created habitats [101]. *Anopheles gambiae* is highly anthropophilic and prefers to feed in human dwellings [98]. After blood feeding, the mosquito rests on interior walls for several hours with limited mobility and can be easily collected [102]. The *Aedes aegypti* mosquito has evolved to specialize in biting humans, and is a possible species for sampling human viruses in tropical Africa and Southeast Asia [103,104]. Mosquito species of the genus *Culex* are distributed in most parts of the world. For example, *Culex erythrothorax* or *Culex quinquefasciatus* mosquitoes feed on various mammals and birds and can ingest viruses from many different hosts [98,105].

Depending on field conditions and available resources, sampled mosquitoes can be stored frozen in liquid nitrogen or dry ice [11,13,14], preserved in RNA-stabilizing buffer such as RNA-later^®^ [106] or blood meals can be preserved on Flinders Technology Associates (FTA) filter paper cards [9,33,37]. For whole mosquitoes, viral particles can be concentrated and purified during sequential processing, including homogenization, filtration, centrifugation and DNA digestion [11,107,108,109]. The average blood meal volume of mosquitoes is 2 µL (1 µL of serum) [110,111,112]. Extracted from FTA cards, this volume is suitable for recovering virus sequences from mosquitoes that fed on artificial blood [9,33,37]. Furthermore, blood meals dried on FTA cards can be maintained at room temperature for weeks prior to NGS without needing cold chains for transport and storage, which is important for sampling under remote field conditions or in underdeveloped countries [113,114]. The denaturation and inactivation of viral particles is provided for and makes the handling of FTA-dried blood spots safe, as shown by the complete inactivation of highly pathogenic Avian influenza virus (AIV) one hour after adsorption onto FTA paper [115]. However, FTA cards cannot be used for viral metagenomics or further analyses of virus samples as cell culture inoculations. Therefore, storing mosquito blood meals on FTA cards is a suitable approach for surveying the viral diversity of a given species, neglecting the value of viral isolates and in vitro culture techniques.

## 5. Discussion

VEM has had its concept proven in several publications. Independent feasibility studies have shown that DNA as well as RNA viruses can be identified from mosquitoes fed on artificial blood or infectious animal models [9,33,37]. Depending on the virus species, the 50% endpoint detection of virus copies/mL is within the natural clinical ranges of human infections [40,41,42,43]. Viral sequences can be detected from the mosquito blood meal for up to 24 h [9,37]. The mosquito midgut is a highly proteolytic environment facilitating the degradation of the blood contents over approximately the first 24 h after blood ingestion [116]. However, particles of human immunodeficiency virus (HIV) even remained infectious for up to ten days in the blood meals of arthropods, including ticks and mosquitoes [117]. VEM was performed successfully in the field, and sequences of human and animal viruses were detected, none of which were able to be vectored or to replicate in the mosquito [9,11]. Furthermore, the detection of a canine virus from a mosquito blood meal by using VEM was linked to the detection of canine host DNA [9]. This combines VEM with iDNA, a term for the genetic material ingested by invertebrates feeding on vertebrates [77].

Although VEM is an innovative and promising approach for sampling and surveying viruses from human and wildlife, one of the main challenges will be to estimate the risk for human health based on the viral sequences found in the blood meal. Since most of these sequences will be “novel”, poorly characterized, incomplete and of unknown pathology, the pathogenicity can hardly be verified by conventional methods. Fredericks et al. and Mokili et al. recently have adapted Koch’s postulates to molecular and NGS data [118,119]. Rather than isolation of the pathogen in cell culture, the metagenomic Koch’s postulates focus on the identification of “metagenomic traits” as sequence reads or contigs that distinguish metagenomes obtained from healthy donors from those of diseased subjects. As an example, the Merkel cell polyomavirus was identified as the causative agent of Merkel’s cell carcinoma [120].

Considering these main pitfalls of VEM, the identification of uncharacterized viral sequences and the missing link to pathology and pathogenicity of these sequences, we suggest that VEM should focus on two different goals. To accelerate the identification of the whole viral diversity, it is necessary to sample a broad range of vertebrates and invertebrates at different places all over the world. Most viruses identified in the viromes of mosquito blood meals were described as “novel” (<70% amino acid identity) [10,36]. These findings suggest that the viral diversity in mosquitoes and in virus reservoirs has hardly been explored yet. It is estimated that 60%–99% of the sequences generated in different viral metagenomic studies are not homologous to known viruses, and more than 320,000 viruses still await their discovery [109,110,111]. Because bioinformatics methods mainly use reference-based approaches for sequence identification, they have so far been unable to classify such highly divergent viruses. Cataloguing the viral diversity of different hosts by VEM can contribute to such reference-based approaches, as it might provide the missing sequence link, which can help classifying viral sequences previously described as “unknown”. The comparison of catalogued viromes of different hosts over space and time should also focus on changes in the virome composition. As the metagenomic Koch’s postulates cannot be verified by distinguishing metagenomics traits of healthy and diseased hosts from the mosquito blood meals, such changes in the virome could be linked to unusual morbidity or mortality events in human and wildlife.

However, VEM can also be applied to easily survey characterized viruses of known pathogenicity over space and time. Since the linkage of such identified viruses to the host is possible with iDNA approaches, the potential health risk might be identified if certain viruses increase remarkably in a given area or emerge in new areas or novel hosts.

## 6. Conclusions

Climate and ecosystem changes, demographics and human behavior have contributed to the increased emergence and re-emergence of viral pathogens globally [5,7,121]. Therefore, monitoring and surveillance of the viral diversity of wildlife and humans in today’s rapidly changing ecosystems can be the key to predicting EIDs before they spread. Virus surveillance with VEM can provide a novel surveillance approach for the detection and monitoring of pathogens prior to disease outbreak and future epidemics.
